# Lung ultrasound: a promising tool to monitor ventilator-associated pneumonia in critically ill patients

**DOI:** 10.1186/s13054-016-1487-y

**Published:** 2016-10-27

**Authors:** Guyi Wang, Xiaoying Ji, Yongshan Xu, Xudong Xiang

**Affiliations:** 1 Department of Intensive Care Unit, The Second Xiangya Hospital, Central South University, Changsha City, Hunan Province China; 2 Department of General Intensive Care Unit, The Second Affiliated Hospital, School of Medicine, Zhejiang University, Hangzhou, China; 3 Department of Emergency, The Second Xiangya Hospital, Central South University, Changsha City, Hunan Province China

**Keywords:** Intensive care unit, Lung ultrasound, Ventilator-associated pneumonia

## Abstract

Ventilator-associated pneumonia (VAP) is the most frequent intensive care unit (ICU)-acquired infection that is independently associated with mortality. Accurate diagnosis and timely treatment have been shown to improve the prognosis of VAP. Chest X-ray or computed tomography imaging are used for conventional assessment of VAP, but these methods are impractical for real-time measurement in critical patients. Therefore, lung ultrasound (LUS) has been increasingly used for the assessment of VAP in the ICU. Traditionally, LUS has seemed unsuitable for the detection of lung parenchyma owing to the high acoustic impedance of air; however, the fact that the reflection and reverberation in the detection region of the ultrasound reflect the underlying pathology of lung diseases has led to the increased use of ultrasound imaging as a standard of care supported by evidence-based and expert consensus in the ICU. Considering that any type of pneumonia causes air volume changes in the lungs, accumulating evidence has shown that LUS effectively measures the presence of VAP as well as dynamic changes in VAP. This review offers evidence for ultrasound as a noninvasive, easily repeatable, and bedside means to assess VAP; in addition, it establishes a protocol for qualitative and quantitative monitoring of VAP.

**Electronic supplementary material:**

The online version of this article (doi:10.1186/s13054-016-1487-y) contains supplementary material, which is available to authorized users.

## Background

Mechanical ventilation is an important life-saving therapeutic means in the intensive care unit (ICU); however, its common complication, ventilator-associated pneumonia (VAP), is associated with increased mortality, use of antimicrobials, mean duration of mechanical ventilation, and healthcare costs [1, 2]. In developing countries, VAP occurs in up to 30 % of critically ill patients on mechanical ventilation and the mean rate of VAP varies from 10 to 41.7 cases per 1000 ventilator days [3–6]. In addition, VAP remains a leading cause of death in hospitalized patients. The mortality related to VAP varies considerably in different kinds of patients, with an approximate mortality rate of 16–94 % in developing countries [5]. The additional financial cost of a VAP episode has been estimated to be more than USD 40,000 [1, 7]. Inappropriate or delayed treatment of VAP can increase the mortality rate compared with when adequate therapy is provided (63.5 versus 29.2 %) [8]. The uncertainties regarding the most appropriate diagnostic method to identify VAP compromise the management of this condition [9]. Therefore, early surveillance and accurate diagnosis remain the cornerstone to ensure the appropriate use of antimicrobial agents and to benchmark the rate of VAP. There is a pressing need to develop reliable monitoring and diagnostic tools for VAP in order to start treatment promptly.

### Diagnosis of VAP

VAP is defined as a new or progressive and persistent radiographic abnormality with evidence of infection or worsening, occurring at least 48 h after the initiation of mechanical ventilation [10]. Thus far, however, there is no consensus regarding the diagnostic criteria for VAP. The current diagnostic criteria for VAP incorporate the symptoms, inflammation bio-indicators, imaging changes, and worsening lung aeration function (Table 1) [11–14]. It is noteworthy, however, that the incidence of VAP varies widely according to the diagnostic criteria. A previous study has reported that the incidence of VAP in the same patient population varied from 4 to 42 % by applying the different diagnostic criteria listed above [15].

**Table 1:** Published diagnostic criteria for VAP

VAP criteria	Inflammatory marks	Sputum	Chest radiography/LUS	Microbiologic or histopathology marks	PEEP/FiO_2_
CDC criteria [11]	Temperature >38 °C, or >36 °C, or WBC ≥12,000 or ≤4000 cells/mm^3^ and new antimicrobial agent is started for ≥4 days	Purulent respiratory secretions		Microbiologic quantitative-positive Endotracheal aspirate ≥10^5^ CFU/mL Broncho-alveolar lavage ≥10^4^ CFU/mL Protected specimen brush ≥10^3^ CFU/mL or histological-positive Lung tissue ≥10^4^ CFU/g or positive for *Legionella*, influenza virus, RSV, adenovirus, or parainfluenza	After a period of stability or improvement on the ventilator, Minimum daily FiO_2_ increase to 0.20 remain for 2 d or daily PEEP values increase to 3 cm H_2_O
CPIS (a score of 6 is suggestive of VAP) [12]	Temperature 38.5–38.9 °C = 1 point; ≥39 or <36.5 °C = 2 points WBC <4000 or >11,000/mm^3^ = 1 point	Non-purulent respiratory secretions = 1 point; purulent respiratory secretions = 2 points	Chest radiography Diffuse infiltrate = 1 point Localized infiltrate = 2 points Progressive infiltrate (without cardiac disease or ARDS) = 2 points	Moderate or heavy microbiologic quantitative or heavy microbiologic quantitative-positive = 1 point Microbiologic quantitative-positive and same pathogenic bacteria seen on Gram stain = 2 points	PaO_2_/FiO_2_ ≤ 240 without ARDS = 2 points
CEPPIS (a score of 5 is suggestive of VAP) [12]	Temperature 38.5–38.9 °C = 1 point; ≥39 or <36.5 °C = 2 points Procalcitonin (ng/mL) ≥0.5 and <1 = 1 point; ≥1 = 2 points	Non-purulent respiratory secretions = 1 point; purulent respiratory secretions = 2 points	LUS-positive (sub-pleural echo-poor region or more with tissue-like echo texture) = 2 points	Microbiologic culture-positive = 2 points	PaO_2_/FiO_2_ ≤ 240 without ARDS = 2 points
CHEST [13]	Temperature >38 °C WBC <4000/mc^3^ or >12,000/mm^3^	Purulent respiratory secretions	Chest radiography New or progressive consolidation		
Johanson criteria [14]	Temperature >38 °C WBC <12,000/mc^3^	Purulent respiratory secretions	Chest radiography New or progressive radiographic infiltrate		

*ARDS* acute respiratory distress syndrome, *CDC* Centers for Disease Control and Prevention, *CEPPIS* Chest Echography and Procalcitonin Pulmonary Infection Score, *CHEST* American College of Chest Physicians, *CPIS* Clinical Pulmonary Infection Score, *FiO*_*2*_ fraction of inspired oxygen, PaO_2_, *PEEP* positive end expiratory pressure, *RSV* respiratory syncytial virus, *WBC* white blood cell

Typically, pneumonia causes air volume changes in the lungs, which mainly reflect as lung consolidation in chest X-ray (CXR) or computed tomography (CT) imaging. More than 90 % of critically ill patients on mechanical ventilation who die in the ICU have histological evidence of lung parenchymal infection [16]. Hence, the conventional VAP algorithms emphasize the radiological findings listed in Table 1. The changes in radiological findings are intrinsic to most diagnostic VAP algorithms [17]. However, evidence suggests that the inherent subjectivity and shortcomings of the CXR findings make it unsuitable for use in the ICU [18, 19]. In response to this clinical dilemma, the National Healthcare Safety Network/Centers for Disease Control and Prevention (CDC) developed a new criterion to improve the objectivity and reproducibility of VAP diagnosis in early 2013 [11]. In the newest CDC criteria, radiographic changes have been replaced by changes in the minimum positive end expiratory pressure (PEEP) and the fraction of inspire oxygen (FiO_2_), which also indirectly reflect air volume changes in the lungs. The change in minimum PEEP or FiO_2_ provides a quantitative and objective metric for the monitoring and diagnosis of VAP. However, some clinical studies have found considerable differences in surveillance between the novel CDC criteria and conventional VAP criteria. A prospective cohort study conducted by Klein Klouwenberg et al*.* [20] found that a maximum of 32 % of VAP cases identified by traditional surveillance satisfied the new criteria. The new criteria show poor concordance with conventional VAP surveillance, making them a confusing surrogate for the quality of ICU care. To the best of our knowledge, this difference in surveillance could be attributed to the replacement of radiographic abnormalities by changes in minimum PEEP or FiO_2_. In addition, a delay in diagnosis along with the increasingly stringent criteria could result in an increased mortality rate associated with VAP from 50 to 80 % [15]. Therefore, a means or device that enables the direct observation and quantification of changes in lung volume would provide a useful and objective metric for the monitoring and diagnosis of VAP.

### Comparison of imaging tools to monitor VAP in the ICU

Currently, international guidelines recommend CXR as a routine evaluation method for suspected pneumonia in an adult as it is a simple technique that enables a rapid diagnosis and provides treatment guidance [21]. However, CXR has some limitations for the diagnosis of pneumonia in the ICU. First, CXR findings may be negative in patients in the early stages of pneumonia or if the pneumonia is present at a location where it is difficult to detect. Moreover, CXR cannot sensitively detect lung consolidations of less than 1 cm [22]. Second, CXR has a low sensitivity and a relatively low accuracy [23]. Butler et al*.* [18] evaluated the use of CXR in detecting VAP in critically ill patients and observed that CXR had a diagnostic sensitivity of only 25 %, a specificity of 75 %, and an accuracy of 45 % when compared with the protected specimen brush technique. Other research also has demonstrated that a normal CXR does not exclude the diagnosis in bedridden patients with suspected pneumonia [24]. Third, the outcome of radiological findings requires some degree of subjective interpretation. Fourth, repeated CXR could overexpose some critically ill patients in the ICU to radiation. These findings suggest that CXR is of limited value for the diagnosis of pneumonia in patients receiving mechanical ventilation in the ICU. It is well known that CT imaging is a better alternative than CXR as it allows visualization of much smaller pulmonary abnormalities [25]. This chest imaging technique offers the highest diagnostic accuracy for detecting pneumonia [26]. However, it cannot be routinely used in all patients suspected of pneumonia owing to its limitations of higher radiation exposure than CXR, the requirement of more medical assistance, and the risk involved in transportation to a CT unit [27]. In this context, there is a need for a nonirradiating, noninvasive, easily repeatable, and bedside method to measure lung volume changes in the ICU.

Lung ultrasound (LUS) originally seemed to be unsuitable for the detection of lung parenchyma because, unlike X-rays, it is unable to cross the underlying air-filled anatomical structures to generate a density-related image. Therefore, LUS was mainly used for the diagnosis and guided puncture of pleural effusion. With advances in ultrasound technology and research in recent years, the advantages of LUS have been gradually realized by utilizing varying absorption, reflection, and reverberation patterns of ultrasound for different interfaces of the lungs. The success of LUS in the diagnosis and monitoring of community-acquired pneumonia (CAP) has been successfully applied to VAP [28]. A multicenter prospective study demonstrated that LUS is a reliable tool for the bedside diagnosis of VAP [29]. A Chest Echography and Procalcitonin Pulmonary Infection Score, a new score based on LUS results and procalcitonin levels, of >5 points was significantly better in predicting VAP than a Clinical Pulmonary Infection Score, based on CXR imaging and white blood cell count, of >6 points [12]. In addition to its excellent diagnostic potential, LUS has advantages, such as high learnability, good diagnostic agreement, and reduced radiation exposure. Moreover, it is a simple technique and requires less sophisticated skills than those required for other sonographic scans (e.g., abdominal or cardiac ultrasound) and the learning curve is faster [30]. In another study conducted by Nazerian et al. [31], LUS showed a good inter-observer variability (k = 0.83) for the diagnosis of lung consolidations when compared with CT. In LUS, the image interpretation itself is less dependent on the operator, which reduces the degree of subjective interpretation [32]. The use of bedside LUS led to a 26 % reduction in CXR and a 47 % reduction in CT scans in the ICU, which, in turn, reduced the exposure of critically ill patients to radiation [33]. Therefore, LUS was recommended as a standard of care by evidence-based and expert consensus to monitor pulmonary infections without the risk of exposure to radiation and the need for transportation from the ICU [34].

### Basic applications of LUS in the ICU

LUS, a recent advance in the ICU, provides direct access to the majority of the lung surface. To achieve a standardized and repeatable outcome, some principles in the application of LUS need to be considered [35]. The first principle is the gravity rule, which helps to determine the location of lung lesions. According to this rule, gas flows toward the sky and fluids flow toward the earth. Thus, the LUS examination for the pneumothorax should focus on the anterior parasternal line, while that for pleural effusion should focus on the posterior axillary line because air flows to nondependent regions but pleural effusion flows to dependent regions in a supine patient. The second principle is the frequency rule, which helps to choose the appropriate detection frequency for different lesions. According to this rule, the frequency of sound waves is negatively correlated with the depth of detection. The high-frequency (5–12 MHz) wave is most effective in visualizing the chest wall, pleura, and the lung peripheral parenchyma, while the 3–5-MHz wave mainly helps to visualize the deeper lung structures. The third principle is the reproducibility rule. In each examination, standardized thoracic points, such as the six spots of electrocardiography, should be defined initially to ensure reproducible analyses.

**Fig. 1:**
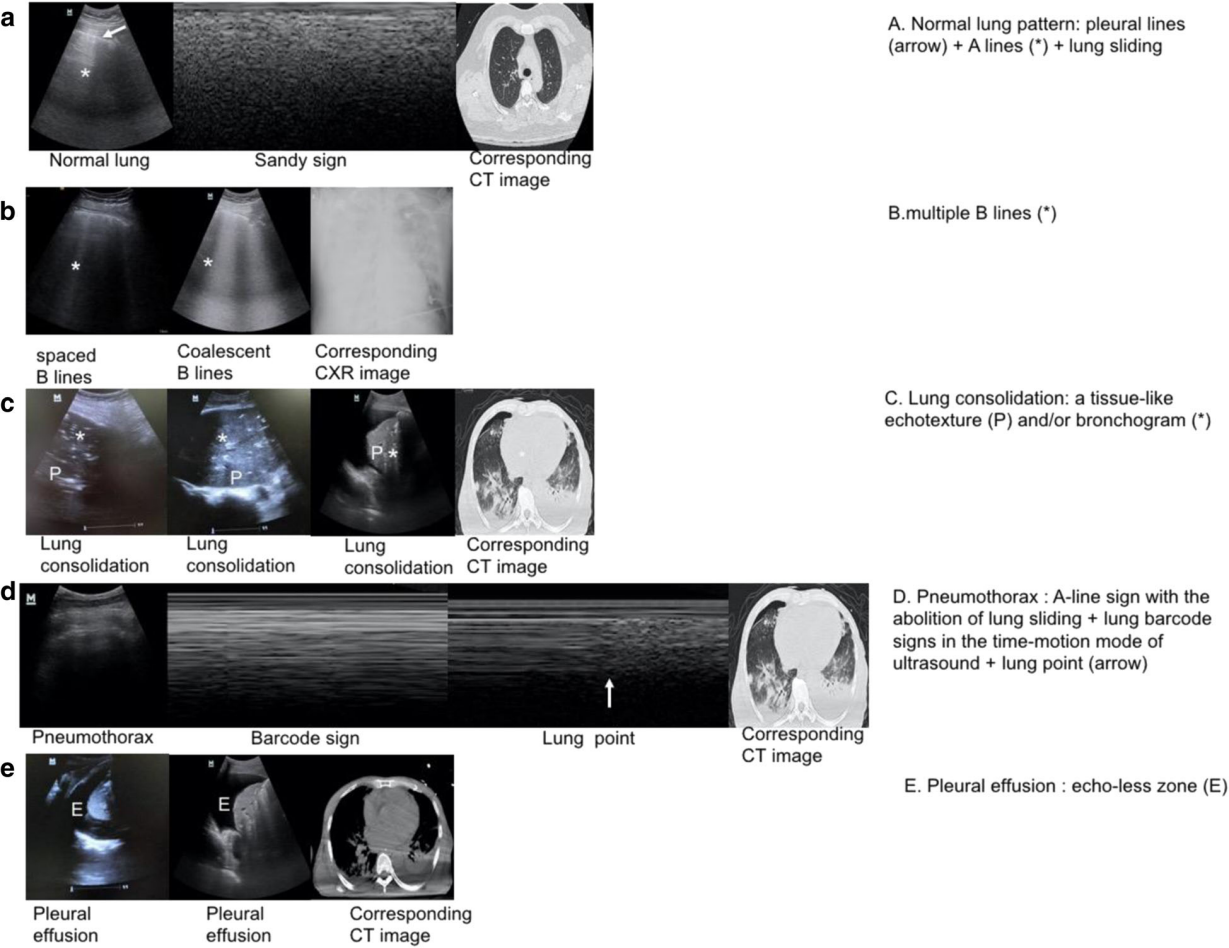
Basic characteristic sonographic patterns. The basic characteristic sonographic patterns are illustrated (*left*) and described according to distinctive features (*right*). **a** A lines are characteristic lines parallel to the pleural line. **b** B lines are long, vertical, hyperechoic, and dynamic lines originating from the pleural line, moving with lung sliding. **c** Lung consolidation is a tissue-like echotexture of the lung with or without a bronchogram. **d** The lung point is a point of contact between normal lung sliding (*sandy sign*) and the absence of lung sliding (*barcode sign*). **e** Pleural effusion is an echo-free zone (*P* tissue-like echotexture of the lung, *E* pleural effusion)

It is well known that the lung is made up of air-filled pulmonary alveoli, spaced with interlobular septa and water. The lung aeration and air/liquid ratio beyond the parietal pleura influence the reflection and reverberation of ultrasound waves, further determining the LUS images of the imaged area. Therefore, the real-time LUS image can indicate the underlying pathology of lung diseases [36]. For quick mastery of LUS in clinical practice, LUS images are summarized to characteristic sonographic patterns according to the lung pathology (Fig. 1).

**Fig. 2:**
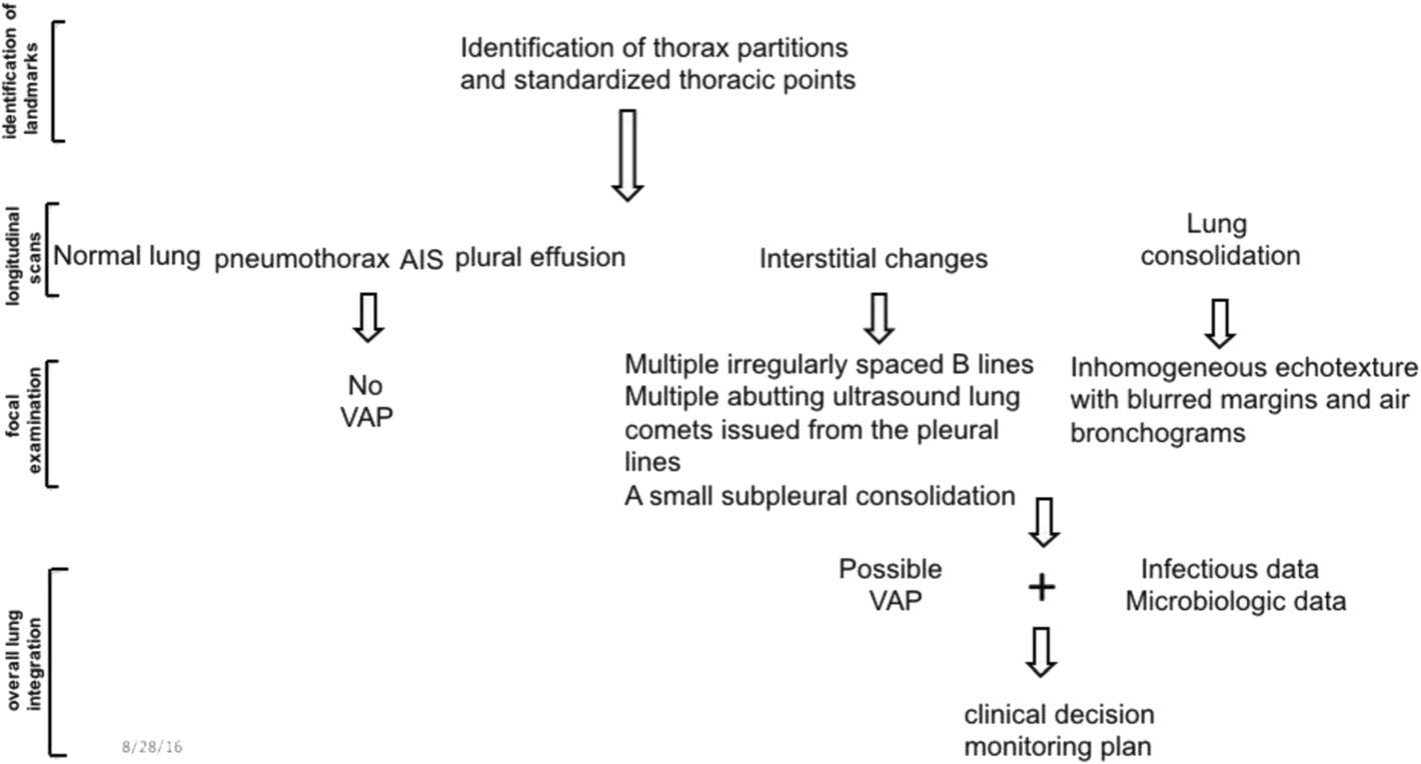
Sequential interpretation of the LUS protocol for detecting VAP. This is a schematic, simplified decision tree of the VAP protocol. The basic steps include the identification of landmarks, longitudinal scans, focal examination, and overall lung integration. The identification of landmarks is helpful for standardized and reproducible analyses. The longitudinal scans mainly provide a preliminary view of the sonographic patterns. The focal examination mainly reveals the characteristic features of a lesion. The overall lung integration enables the translation of all data into a possible clinical decision and monitoring plan. *AIS* alveolar interstitial syndromes, *VAP* ventilator-associated pneumonia


Typically, the surfaces of the visceral and parietal pleurae slide forward and backward against each other during respiration. Ultrasound waves are reflected by the interface of the pleurae, which generate a hyperechoic, sliding line termed the pleural line with lung sliding (Fig. 1a). In addition, using the time-motion mode of ultrasound, lung sliding below the pleural line appears as a homogenous granular pattern called a sandy sign (Fig. 1a). On the far side of the pleural line, the lungs generate linear images called A lines, which are parallel to the pleural line because of the high acoustic impedance of air, wave reflection, and reverberation. The white and hyperechoic A lines, which become less intense with depth, are static and appear at regular intervals (Fig. 1a). The combination of a pleural line, bilateral lung sliding, and an A line indicate normal lung aeration (Fig. 1a; Additional file 1: Video S1). It is noteworthy, however, that a normal lung pattern can be observed in those with emphysema or asthma. The B line is a long, vertical, hyperechoic, and dynamic line that originates from the pleural line, moves with lung sliding, and spreads to the edge of the screen without fading the A lines (Fig. 1b; Additional file 2: Video S2). B lines represent a reverberation artifact through thickened subpleural interlobular septa by deposition of fibrous tissues, inflammatory cells, or pulmonary edema [37]. Thus, a B line can be detected in cardiogenic pulmonary edema, acute respiratory distress syndrome (ARDS), interstitial lung diseases, pneumonia, etc. [37, 38]. The presence of multiple vertical B lines more than 7 mm apart (spaced B lines) indicates a moderate decrease in lung aeration due to thickened interlobular septa. The presence of coalescent B lines less than 3 mm apart (alveolar interstitial syndromes) indicates a more severe decrease in lung aeration due to partial filling of alveolar spaces [28]. It should be noted that detection of fewer than three isolated B lines in dependent regions of a normal lung could have no pathological significance [39]. Lung consolidations are characterized by a tissue-like echotexture similar to that observed in liver parenchyma on LUS (Fig. 1c; Additional file 3: Video S3). They are detected in pneumonia, lung atelectasis, lung contusion, ARDS, etc. The presence of lung consolidation indicates complete loss of lung aeration because the alveolar space is filled with exudates and cellular debris or is collapsed by the proliferation of neoplastic tissue or pleural effusion [40]. Within the consolidation, a branch-shaped or horizontal bronchogram containing air or fluid can be observed (Fig. 1c). In such cases, an inspiratory reinforcement corresponding to penetration of air into the bronchial tree, called a dynamic air bronchogram, can be occasionally observed [41]. The pneumothorax is characterized by the “lung point”, disappearance of lung sliding, and the “barcode sign” in the time-motion mode on LUS (Fig. 2b). The lung point is a characteristic marker of the pneumothorax (Fig. 1d) [42]. It is the transition between normal lung sliding and the absence of lung sliding at the pneumothorax border. By using the time-motion mode of ultrasound, the absence of lung sliding appears as strictly horizontal lines, called the barcode sign (Fig. 1d) [43]. The absence of lung sliding or B lines is helpful to exclude the pneumothorax with a 100 % negative predictive value because these two signs indicate the movement of intact visceral pleura against parietal pleura [43]. Lung effusion appears as an echo-free zone on LUS, which helps to differentiate the presence of fluids or consolidation, leading to the opacity of the hemithorax in ICU patients (Fig. 1e; Additional file 4: Video S4). It is noteworthy that lung effusion is usually accompanied by another condition such as pneumonia or pulmonary edema. Thus, understanding the etiology of pleural effusion through LUS is important.

Additional file 1:Normal lung aeration. (AVI 4812 kb)
Additional file 2:B lines. (AVI 4411 kb)
Additional file 3:Lung consolidations. (AVI 2898 kb)
Additional file 4:Lung effusion. (AVI 5546 kb)


### Applications of LUS in CAP and VAP

LUS has the advantages of good diagnostic efficiency and real-time monitoring for CAP and VAP. In a study of 179 patients that compared the accuracy of LUS and CXR for the diagnosis of CAP, LUS was found to be better than CXR (sensitivity of 94.6 versus 77.7 % and accuracy of 96.1 versus 83.8 %, respectively; *p* < 0.001) [44]. In addition, Bourcier et al. [45] compared the duration of symptoms with the respective performance of LUS and CXR for the diagnosis of CAP and observed that LUS detected more cases of CAP compared with CXR in the first 24 h of care (76 versus 23 %). This result further suggested that LUS was more sensitive than CXR in the early diagnosis of CAP [45]. During the follow-up of patients with CAP, LUS could effectively monitor the changes in the lesion area. In a multicenter study to define the accuracy of LUS, the median area of pneumonic lesions in patients decreased from 15.3 to 0.2 cm^2^ on days 13 to 15 compared with the baseline. This change was in line with the change in the median C-reactive protein levels of patients along with their improved condition [46]. For VAP, the sensitivity and specificity of LUS in detecting lung consolidation were 90 and 98 %, respectively, with CT as a gold standard [41]. Because the nature of CAP or VAP is pneumonia induced by pathogens such as bacteria or viruses, the two types of pneumonia possess similar pathological features, resulting in homogeneous ultrasound features. The core feature of VAP or CAP observed on LUS is lung consolidation accompanied by an air bronchogram [41, 46]. However, LUS is more convenient for the diagnosis of CAP than VAP because patients with CAP are more cooperative and seldom have thoracic dressings or drainage tubes.

### Diagnosis and management of VAP

Mechanically ventilated patients often show a wide range of abnormal patterns on LUS. The sonographic features of VAP can be easily confused with those of lung cancer, pulmonary embolism, atelectasis, etc., leading to a 17–26 % rate of misdiagnosis in the critical care setting [47]. Thus, it is important to establish a protocol for the accurate diagnosis and monitoring of VAP. This protocol is required for qualitative and quantitative examination of the lung with panoramic and dynamic views, including an understanding of the anatomy, pathophysiology, and clinical signs of VAP (Fig. 2).

First, panoramic evaluation is achieved by the identification of landmarks, longitudinal scans, focal examination, and overall lung integration [36]. The following landmarks should be identified: the anterior parasternal line, anterior axillary line, and posterior axillary line, which divide each hemithorax into anterior, lateral, and posterior areas, respectively [41, 48]. The anterior and lateral lung regions of the patients in the ICU are usually evaluated in a supine position and the posterior region is evaluated in the lateral decubitus position. Every region of the hemithorax has its own significance of detection on LUS. Pneumothorax or interstitial syndrome can be detected in the anterior chest wall, while for the diagnosis of VAP, LUS of the lateral and posterior areas, rather than the anterior wall, is performed [41]. The longitudinal scans are performed from the clavicle to diaphragm (mammillary line or one or two intercostal spaces below), along the intercostal spaces. The longitudinal scanning data provide a preliminary view of the sonographic patterns. A focal examination scans the area of interest for the accurate identification of the lesion with different scans (longitudinal, transverse, and oblique views) and, sometimes, also with different probes. Subsequently, all data from the longitudinal scans and focal examination are translated into a possible clinical decision and monitoring plan by overall lung integration.

Second, a dynamic and continuous progression from normal aeration to complete loss of aeration exists in VAP. When lung aeration is disturbed because of VAP, a normal LUS pattern gradually changes from the appearance of focal areas of interstitial syndrome (spaced B lines, which become confluent B lines) to subpleural small consolidations. These subpleural consolidations can further develop into lobar consolidations [49]. Thus, the sonographic features of VAP vary according to the development of the lesion and are determined according to the distribution characteristics, interstitial changes, and parenchymal changes [41]. The interstitial changes of VAP comprise multiple irregularly spaced B lines, multiple abutting ultrasound lung comets arising from the plural lines, and a small subpleural consolidation [28]. The parenchymal characteristic of VAP is lung consolidation of an inhomogeneous echotexture with blurred margins and air bronchograms [28, 50, 51]. Among these characteristics, air bronchograms are an effective marker for the diagnosis of VAP, with a sensitivity of 100 % and a specificity of 60 % [50]. However, there is a need to differentiate the nature of lung consolidations during focal examination because the lesion may be pneumonia, lung atelectasis caused by mechanical obstruction or compression, tumor consolidation, pulmonary embolism, etc. For example, lung atelectasis is characterized by regular margins and no dynamic air bronchograms. The feature of pulmonary embolism is a wedge-shaped, hypoechoic consolidation, typically in the dorsal and basal regions of the lung. However, these features of B-mode ultrasound are not sufficiently specific for the differential diagnosis of VAP. The vascular pattern within the consolidation, as assessed by color Doppler ultrasound, provides an alternative means for determining the etiology of pulmonary consolidations (Table 2) [52, 53]. The vascular pattern indicators, including the pulsatility index, the resistance index, and the duration between the initial and peak systolic velocity, have been reported to be useful for differentiating between the consolidations of different etiologies [52]. These results imply that blood flow of lung atelectasis caused by mechanical obstruction is a high-impedance flow, pneumonia is a moderate-impedance flow, tumor consolidation is a low-impedance flow, and pulmonary embolism is without any blood flow. The discrepancy in hemodynamic changes observed in the regional pulmonary artery in lung consolidation may be mainly attributed to reactive vasoconstriction due to local hypoxia [54]. An air bronchogram is a specific sign for the diagnosis of VAP, which implies that the resulting reactive vasoconstriction to hypoxia is less, because air can still enter the partially filled alveoli during inspiration. In lung atelectasis caused by mechanical obstruction, the complete airway obstruction accounts for regional hypoxia and severe reactive vasoconstriction. Neovascularization is a characteristic feature of tumor consolidation, which accounts for the low-impedance flow. However, other researchers also have found that pulmonary blood flow persists within the consolidations caused by ARDS or a diffuse alveolar hemorrhage [55, 56]. These results indicate that the hemodynamic changes observed in the regional pulmonary artery during lung consolidation are complex. However, this phenomenon was consistent with the increased pulmonary blood flow observed in the damaged regions of an ARDS rat model induced directly by acid aspiration because the relaxing factors released from the damaged alveoli limit hypoxic pulmonary vasoconstriction [57–59].

**Table 2:** Spectral waveform analysis of pulmonary arterial flow patterns in different etiologies

	Pneumonia	Lung atelectasis	Tumor consolidation	Pulmonary embolism
Presence of flow signal	Detected	Detected	Detected	None detected
Flow signal density	High	Low	Low	None
PI ([Peak systolic velocity − End diastolic velocity]/Mean velocity)	Median	High	Low	None
RI ([Peak systolic velocity − End diastolic velocity]/Peak systolic velocity)	Median	High	Low	None
AT (duration from the beginning to the peak systolic velocity)	Median	Low	High	None
The feature of blood flow	Moderate-impedance flow	High-impedance flow	Low-impedance flow	No flow

Third, LUS has been successfully applied for the early diagnosis of VAP. In a multicenter prospective study that included 99 patients suspected of VAP, the combined LUS features of subpleural consolidation and air bronchograms showed a positive predictive value of 86 % (ClinicalTrials.gov NCT02244723) [29]. However, the clinical diagnosis of VAP should not be based on the LUS image alone but on the combination of LUS findings, clinical parameters, and microbiological results. This combination approach increases the diagnostic accuracy of LUS [29].

Lastly, LUS scoring systems based on the A line, B line, and lung consolidation have been applied for the quantitative monitoring of lung aeration and aeration changes [28, 55, 60, 61]. All these scoring systems divide lung aeration into four ultrasound patterns for the assessment of aeration and re-aeration observed on LUS. The four ultrasound patterns include normal aeration (N; the presence of lung sliding with A lines or fewer than two isolated B lines), moderate (B1; multiple well-defined B lines), severe (B2; multiple coalescent B lines), and complete (C; the presence of a tissue pattern characterized by dynamic air bronchograms). The differences among these scoring systems mainly lie in the magnitude and changes of ultrasound patterns (Table 3). The accuracy of LUS in assessing lung aeration in VAP has been demonstrated by constructing an LUS-based scoring system [28]. A tight correlation was observed between the change in the LUS-based scoring system and CT measurements of lung aeration after antimicrobial therapy (day 0 versus day 7). An ultrasound score >5 demonstrated a CT re-aeration >400 mL and successful antimicrobial therapy. Thus, LUS is an effective tool to turn images into numbers (semi-quantification) for evaluating the status of VAP and the effect of therapy.

**Table 3:** Comparison among different lung aeration scores

	Lung aeration score		Lung aeration change score (re-aeration/loss of aeration)	
Application object	Lung aeration evaluation to predict post extubation distress [60] Lung aeration evaluation to patients with ARDS [55]		PEEP-induced lung aeration changes in patients with ARDS [61] Antibiotic-induced lung aeration changes in patients with VAP [28]	
Value	N B1 B2 C	0 point 1 point 2 points 3 points	N ↔ B1; B1 ↔ B2; B2 ↔ C N ↔ B2; B2 ↔ C B2 ↔ C	1 point 3 points 5 points

### Limitations of LUS in VAP

Despite the ease of use, bedside availability, noninvasiveness, and repeatability of LUS, this technique may not be suitable for obese patients with a thick chest wall, patients with pleural calcifications, noncooperative patients, and patients with thoracic dressings or a drainage tube. In addition, about 20 % of the lung surface is not visualized by LUS owing to the shielding of anatomic structures (clavicle and scapula) [48]. The detection efficiency of LUS for VAP is also influenced by the lesion size and by the distance between the lesion and lung surface. Small consolidations measuring less than 20 mm that are located posteriorly and away from the pleura may not be detected by LUS [41]. In addition, there is a need for adequate training among clinicians who are unfamiliar with the use and interpretation of ultrasound images. Furthermore, LUS cannot be considered disease specific. It should always be combined with the patient history, physical examination, and laboratory analysis.

## Conclusions

Published data show that LUS is an accurate bedside tool to detect and monitor VAP, especially in the critical care setting. It helps to reduce the overexposure of patients to radiation. Therefore, the use of LUS as a standard of care should be encouraged, especially in the ICU. However, the presence of specific sonographic features merely indicates VAP. Further research regarding a protocol including LUS findings as well as infectious and microbiological data is warranted to increase the diagnostic efficiency of VAP in clinical practice.

## Abbreviations

ARDS: Acute respiratory distress syndrome
CAP: Community-acquired pneumonia
CDC: Centers for Disease Control and Prevention
CT: Computed tomography
CXR: Chest X-ray
FiO_2_: Fraction of inspired oxygen
ICU: Intensive care unit
LUS: Lung ultrasound
PEEP: Positive end expiratory pressure
VAP: Ventilator-associated pneumonia
